# Psychometric Properties of the RESTQ-Sport-36 in a Collegiate Student-Athlete Population

**DOI:** 10.3389/fpsyg.2021.671919

**Published:** 2021-05-26

**Authors:** Stacy L. Gnacinski, Barbara B. Meyer, Carly A. Wahl

**Affiliations:** ^1^Department of Health Sciences, Drake University, Des Moines, IA, United States; ^2^Laboratory for Sport Psychology & Performance Excellence, Department of Rehabilitation Sciences & Technology, University of Wisconsin-Milwaukee, Milwaukee, WI, United States

**Keywords:** training load monitoring, validity, reliability, stress, recovery

## Abstract

The purpose of the current study was to examine the reliability and validity of the RESTQ-Sport-36 for use in the collegiate student-athlete population. A total of 494 collegiate student-athletes competing in National Collegiate Athletic Association Division I, II, or III sanctioned sport completed the RESTQ-Sport-36 and Brief Profile of Mood States (POMS). Structural equation modeling (SEM) procedures were used to compare first order to hierarchical model structures. Results of a confirmatory factor analysis (χ^2^[528] = 1129.941, *p* < 0.001; SRMR = 0.050; CFI = 0.929) and exploratory structural equation modeling analysis (χ^2^[264] = 575.424, *p* < 0.001; SRMR = 0.013; CFI = 0.963) indicated that the first order 12-factor structure demonstrated the best fit of all models tested. Support was not observed for the fit of any hierarchical model. Moderate to strong correlations were observed between stress and recovery subscales and mood states, thus supporting the construct validity of the abbreviated RESTQ measurement model. The current findings provide support for the measure’s use in this population and give pause as it relates to the scoring and interpretation of hierarchical factors such as *Total Stress* and *Total Recovery*. Overall, the current results indicate that the RESTQ-Sport-36 may be a useful tool for collegiate student-athlete training load and competition monitoring.

## Introduction

The contemporary sport performance literature highlights the benefit of incorporating psychological surveys within elite athlete training load monitoring and management protocols (Saw et al., 2016; Schwellnus et al., 2016; Soligard et al., 2016; Bourdon et al., 2017). Among other measures like the Profile of Mood States (POMS), the Recovery Stress Questionnaire for Athletes (RESTQ-Sport, Kallus and Kellmann, 2016) is one of the most frequently used measures for monitoring elite athlete responses to training load (Saw et al., 2016; Kellmann et al., 2018). Across primary research studies, collective evidence indicates that the RESTQ-Sport is sensitive to changes in training load (Kölling et al., 2016; Nicolas et al., 2019), illness or injury risk (Laux et al., 2015; van der Does et al., 2017; Heidari et al., 2018), and performance (Filho et al., 2015; Otter et al., 2016). Despite the strengths of the measure, and the overwhelming popularity of the RESTQ-Sport among researchers and practitioners alike, studies examining the psychometric properties of the original RESTQ-Sport measure have generated conflicting results over the past 20 years (Kellmann and Kallus, 2001; Davis et al., 2007; Martinent et al., 2014; Kallus and Kellmann, 2016).

Concurrent with the widespread popularity of the original 76-item RESTQ-Sport measure among researchers, the authors developed a shortened 52-item version. While some researchers utilized this measure (Tessitore et al., 2011; Kuan and Kueh, 2015; Laux et al., 2015), the 76-item RESTQ-Sport, by far, remained the most used form in research studies. Due to the growing practical concerns regarding the psychometric properties, measure length, scoring procedures, and translational utility of 76-item RESTQ-Sport data to inform interventions aimed at overtraining prevention (Taylor et al., 2012; Saw et al., 2015), the authors recently developed a further abbreviated 36-item version called the RESTQ-Sport-36 (Kallus and Kellmann, 2016). In comparison to the longer versions, the RESTQ-Sport-36 involves a balanced measurement model (i.e., 12 factors, 3 items per factor) while eliminating conceptually redundant factors and items. Using structural equation modeling (SEM) procedures to analyze data from a sample of German athletes, Kallus and Kellmann (2016) reported support for model fit across individual subscales. To the extent of the authors’ knowledge, no examinations of hierarchical models were conducted in this initial validation.

Nicolas et al. (2019) conducted additional research to support the psychometric properties of the RESTQ-Sport-36 among French-speaking athletes. Superior model fit was reported for the 12-factor measurement model (χ^2^ [528] = 1215.36, *p* < 0.001; RMSEA = 0.05; CFI = 0.951), over all hierarchical models. Further, reliability estimates for each of the subscales were interpreted as acceptable, with the exception of the *Disturbed Breaks* and *Social Recovery* factors. Although strong psychometric properties of the measure have been observed among European elite athletes, RESTQ-Sport-36 reliability and validity have yet to be established among English-speaking or United States-based athletes.

It should be noted that neither Kallus and Kellmann (2016) nor Nicolas et al. (2019) found evidence to support the measurement of hierarchical constructs like *Total Stress* or *Total Recovery*, yet these variables are used frequently in research methods. Drawing back to the larger body of RESTQ literature, such discrepancies in scoring, and therefore statistical analysis, may be contributing to the inconsistencies found across studies and/or questions surrounding the practical meaning of findings generated. Critical examinations of the validity of construct measurement across levels within a hierarchical structure like the RESTQ-Sport-36 are necessary, as even a cursory review of the RESTQ literature reveals significant variation in the scoring procedures used by different research teams (e.g., van der Does et al., 2015; Otter et al., 2016). As such, additional investigations are needed to confirm the most valid means of scoring the RESTQ-Sport-36 to advance this area of the literature. Such research would support the translatability of RESTQ-Sport-36 data, advancing this well-established monitoring or “red flagging” tool to one which can inform the design of precise and effective recovery interventions.

Finally, scientists have only recently shifted attention toward monitoring training load among collegiate student-athletes in the United States (Conte et al., 2018; Flatt et al., 2018; Govus et al., 2018; Hamlin et al., 2019; Huggins et al., 2019; Sampson et al., 2019). Previous research has demonstrated that the collegiate student-athlete population may experience elevated levels of stress and burnout due to perfectionistic tendencies, competing demands as student and athlete, insufficient self-regulation skills, as well as responses to training load and competition (Gould and Whitley, 2009; Dubuc-Charbonneau and Durand-Bush, 2015; Garinger et al., 2018; Huml et al., 2019). Other research has noted that the strength of student-athletes academic and athletic identities varies by age and competition levels (Lupo et al., 2017a), and that student-athlete motivation toward a dual-career may be influenced by gender, age, competition level, type of sport, and year of attendance (Lupo et al., 2017b). Such findings support the value of monitoring the stress and recovery experiences of student-athletes, to support their health and well-being amidst progression toward academic, sport, or dual-career goals. Despite this growing need to monitor collegiate student-athlete responses to training load and competition, researchers have yet to apply any of the RESTQ-Sport derivative measures within this population. The RESTQ-Sport-36 specifically could be used as a brief and valid tool to assess collegiate student-athlete internal load, thus adding depth and rigor to the overall training load monitoring protocols already in place. Within a robust United States collegiate student-athlete sample, the purposes of this study were to: (a) examine the reliability and structural validity of the RESTQ-Sport-36 using SEM procedures and (b) examine construct validity of the RESTQ-Sport-36 via correlations between its subscales and mood states as measured by the Brief POMS. An additional applied purpose of the study was to determine best scoring methods for the RESTQ-Sport-36.

## Materials and Methods

### Participants

Participants were solicited via e-mail recruitment flyers, word-of-mouth, and personal invitation through existing collaborations with the second author. Athletes (*N* = 494, mean age = 19.7 ± 1.4, 68.4% female) currently participating in a variety of sports volunteered to participate in the current study. Sample demographics are reported in Table 1. All participants were competing in a National Collegiate Athletic Association (NCAA) Division I, II, or III sanctioned sport at the time of survey completion.

**TABLE 1:** Student-athlete demographic characteristics.

Characteristic	*n*	Percent by category (*N* = 494)
**Gender**		
Male	156	31.6%
Female	338	68.4%
**Race/ethnicity**		
Caucasian/White	434	87.9%
Black/African American	21	4.3%
Latino/a or Hispanic	20	4.0%
Asian	4	0.8%
Native American	2	0.4%
Other	11	2.2%
**Season status**		
Pre-Season or Training Camp	58	11.7%
In-Season	321	65.0%
Off-Season	108	21.9%
Other	6	1.2%
**Competition level**		
NCAA Division III	189	38.3%
NCAA Division II	78	15.8%
NCAA Division I	226	45.7%
**Sports**		
American Football	5	1.0%
Baseball	7	1.4%
Basketball	51	10.3%
Cross Country	29	5.9%
Diving	9	1.8%
Fencing	2	0.4%
Field Hockey	8	1.6%
Gymnastics	7	1.4%
Ice Hockey	19	3.8%
La Crosse	7	1.4%
Rowing	4	0.8%
Soccer	109	22.1%
Softball	47	9.5%
Swimming	103	20.9%
Tennis	7	1.4%
Track and Field	66	13.4%
Volleyball	10	2.0%
Water Polo	1	0.2%
Wrestling	2	0.4%
Other	2	0.4%

*NCAA, National Collegiate Athletic Association. Missing data not included in table.*

### Measures

**FIGURE 1:**
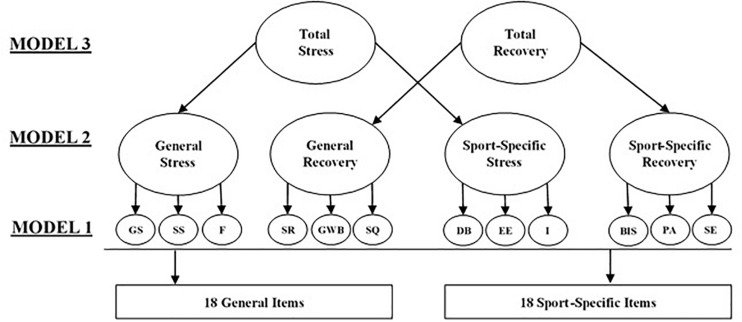
Hypothesized models tested. Covariance between latent variables not depicted.

The RESTQ-Sport-36 (Kallus and Kellmann, 2016) was administered first to each participant. Adapted from the original RESTQ-Sport measure (Kellmann and Kallus, 2001; Kallus and Kellmann, 2016), the hypothesized model consists of 36 items and 12 first-order factors, with three items used to measure each factor. Second-order factors consisted of *General Stress*, *General Recovery*, *Sport-specific Stress*, and *Sport-specific Recovery*. Third-order factors consisted of *Total Stress* and *Total Recovery*. The hypothesized measurement model structures are shown in Figure 1. All items in the RESTQ-Sport-36 begin with the stem of “In the past 3 days/nights,” and athletes indicated item responses on a 7-point Likert scale ranging from never (0) to always (6). Item responses, treated as interval or continuous data, were interpreted as athletes’ perceived frequency of events and behaviors.

The Brief Profile of Mood States (Brief-POMS) was administered to assess athlete mood states (McNair et al., 1992). The Brief-POMS is a 30-item measure, with items scored on a 5-point Likert scale ranging from 0 (not at all) to 4 (extremely). All items reflect descriptions of feelings over the past week. The reliability and validity of the measure for use in adult populations has been established in previous research (McNair et al., 1992; Bourgeois et al., 2010). Calculated as the sum of all items, each mood state score ranged from 0 to 20.

### Procedure

Prior to participant recruitment, study methods were reviewed and approved by the institutional review board at the second author’s affiliate university. Athletes who completed the informed consent to participate submitted their Qualtrics online survey responses between August 2016 and February 2017. Participants completed a demographic survey, the RESTQ-Sport-36, and the Brief-POMS at a location and time of their convenience, amounting to approximately 10–15 min for survey completion. All survey responses were collected anonymously.

### Statistical Analysis

All SEM analyses were performed using Mplus 8.0 software (Muthén and Muthén, 2011). To determine the most parsimonious factor structure of those shown in Figure 1, the hypothesized models were tested in order of structural complexity using confirmatory factor analysis (CFA) procedures. Model 1 represented the first order factor structure, in which 36 items load onto 12 latent variables. Model 2 represented the structure described in Model 1, with the addition of the four second order *General* and *Sport-specific* latent variables. Model 3 represented the structure described in Model 2, with the addition of the two third order *Total Stress* and *Recovery* latent variables.

To account for potential cross-loading across items and to support flexibility in the representation of a complex measurement model such as the RESTQ-Sport-36, an exploratory structural equation modeling (ESEM) procedure was also applied to Model 1. Within the ESEM procedure, a target (orthogonal) rotation was used. Goodness of fit was compared across all models tested to evaluate parsimony. To determine subscale reliability, McDonald’s omega coefficient computations were performed using parameters obtained from both the CFA and the ESEM first order model. Omega coefficients of greater than 0.70 were considered acceptable. Missing data were treated as missing completely at random, and thus treated using full information maximum likelihood estimation for incomplete data procedures (Enders and Bandalos, 2001; Kline, 2011).

The covariance matrix was analyzed using the maximum likelihood (ML) estimation procedure. The covariance matrix utilized is presented within the Supplementary Material. To define units within Model 1, the unstandardized loading of one item from each first order latent variable was constrained to 1.0. For Models 2 and 3, a standardization approach was utilized, whereby the variance of a common factor was constrained to 1.0. Results from previous simulation studies have demonstrated that when items are evaluated by 5 or more categories, data are normally distributed, and adequate sample size is achieved, acceptable model rejection rates are yielded by ML estimation methods (Beauducel and Herzberg, 2006; Rhemtulla et al., 2012). To evaluate the model fit, the chi-square test of fit, residuals-based indices (i.e., root mean square error of approximation [RMSEA], standardized root mean square residual [SRMR]), and incremental fit indices (i.e., comparative fit index [CFI], Tucker-Lewis index [TLI]) were calculated and reported. All calculated model fit indices were collectively evaluated in light of previous literature on model fit determinations (Hu and Bentler, 1999; Kenny and McCoach, 2003; Marsh et al., 2004; Jackson et al., 2009).

Relationships between RESTQ-Sport-36 variables and mood states were examined using Pearson correlation coefficients. Given that this analysis was performed to explore construct validity, statistical significance was not interpreted as meaningful. Rather, correlation coefficient magnitudes of 0–0.3 were interpreted as weak, 0.3–0.7 were interpreted as moderate, and 0.7–1.0 were interpreted as strong. As the Brief POMS was administered after the RESTQ-Sport-36, attrition during survey completion was observed. A significant proportion of athletes did not complete the Brief POMS after completing the RESTQ-Sport-36 (38.5%). As such, listwise deletion was used to ensure that only complete responses from athletes were used in the analysis. A final sample size of 304 was used for the correlation analysis.

## Results

**FIGURE 2:**
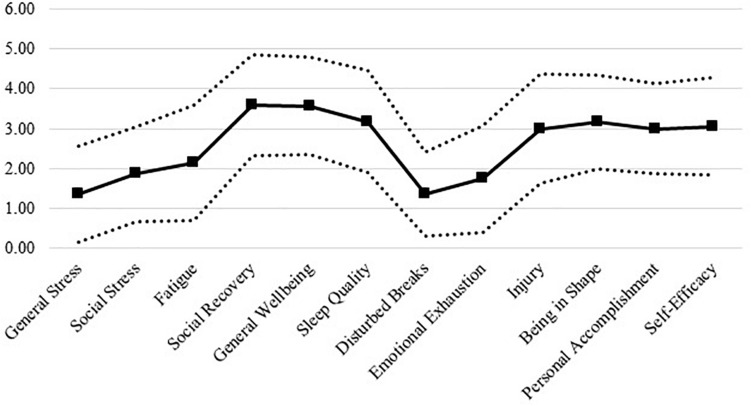
A snapshot stress-recovery profile of collegiate student-athletes.

Descriptive statistics for RESTQ-36-Sport responses are displayed in Figure 2.

Model fit comparisons are presented in Table 2. Measurement model parsimony was observed in Model 1 (χ^2^[528] = 1129.941, *p* < 0.001; SRMR = 0.050; CFI = 0.929), indicating that the first order 12-factor structure is the best fitting model of the three models tested. Support was not observed for the fit of any hierarchical model. This finding was further reinforced by the ESEM model test (χ^2^[264] = 575.424, *p* < 0.001; SRMR = 0.013; CFI = 0.963), which demonstrated improvements in the SRMR and CFI parameters beyond those which were observed in the CFA procedure for Model 1. Model parameter estimates, residual variances, and omega coefficients of the good-fitting models are reported in Table 3. It is worth noting that some standardized factor loadings (e.g., GS3, BIS4, SE2, see) from the ESEM were smaller in magnitude than the standardized factor loadings from the CFA, indicating that the proportion of indicator variance explained by the respective factor is affected by whether or not the model accounts for cross-loading. For example, only 6.6% of the variance in GS3 is accounted for by *General Stress* using the ESEM procedure. All other items loaded appropriately on to the respective factors, and factor loadings and residual variances were consistent with theory. Omega coefficients demonstrated acceptable levels of reliability for all subscales except *Personal Accomplishment* (both CFA and ESEM procedures) and *Being in Shape* (ESEM procedure only). Standardized relationships between first order latent variables from the CFA and ESEM models are displayed in Table 4. The directionality and magnitudes of relationships observed are consistent with the underlying measurement theory (Kallus and Kellmann, 2016).

**TABLE 2:** Model comparisons by fit indices.

	AIC	BIS	χ ^2^ (*df*)	*p*	RMSEA (90% CI)	SRMR	CFI	TLI
Model 3	54770	55291	1453.747 (577)	<0.001	0.055 (0.052–0.059)	0.081	0.897	0.887
Model 2	54741	55265	1424.806 (577)	<0.001	0.055 (0.051–0.058)	0.079	0.900	0.891
Model 1	54499	55231	1129.941 (528)	<0.001	0.048 (0.044–0.052)	0.050	0.929	0.915
ESEM	54168	56009	575.424 (264)	<0.001	0.049 (0.043–0.054)	0.013	0.963	0.912

**TABLE 3:** Model 1 CFA and ESEM comparisons of standardized factor loadings and reliability.

	CFA Standardized λ (*S.E.*)	CFA Residual Variance (*S.E.*)	CFA ω	ESEM Standardized λ (*S.E.*)	ESEM Residual Variance (*S.E.*)	ESEM ω
**General stress**						
GS1	0.855 (0.023)	0.269 (0.039)	0.85	0.834 (0.217)	0.157 (0.092)	
GS2	0.759 (0.030)	0.424 (0.045)		0.750 (0.325)	0.353 (0.072)	0.80
GS3	0.823 (0.024)	0.322 (0.039)		0.257 (0.152)	0.320 (0.033)	
**Social stress**						
SS1	0.889 (0.017)	0.209 (0.030)		0.846 (0.090)	0.191 (0.067)	
SS2	0.924 (0.013)	0.146 (0.024)	0.88	0.887 (0.082)	0.131 (0.046)	0.88
SS4	0.692 (0.034)	0.521 (0.046)		0.587 (0.056)	0.444 (0.049)	
**Fatigue**						
FG2	0.790 (0.028)	0.376 (0.044)		0.769 (0.080)	0.395 (0.067)	
FG3	0.859 (0.024)	0.262 (0.041)	0.84	0.990 (0.075)	0.106 (0.081)	0.85
FG4	0.727 (0.035)	0.471 (0.051)		0.499 (0.081)	0.410 (0.049)	
**Disturbed breaks**						
DB2	0.755 (0.033)	0.430 (0.050)		0.788 (0.221)	0.341 (0.196)	
DB3	0.756 (0.036)	0.429 (0.054)	0.79	0.644 (0.143)	0.388 (0.132)	0.78
DB4	0.716 (0.039)	0.487 (0.056)		0.640 (0.148)	0.503 (0.087)	
**Injury**						
INJ1	0.687 (0.035)	0.528 (0.049)		0.703 (0.073)	0.436 (0.070)	
INJ2	0.731 (0.032)	0.466 (0.047)	0.78	0.552 (0.074)	0.440 (0.074)	0.78
INJ3	0.807 (0.027)	0.349 (0.044)		0.801 (0.185)	0.320 (0.121)	
**Emotional exhaustion**						
EE1	0.764 (0.037)	0.416 (0.056)		0.691 (0.113)	0.341 (0.050)	
EE3	0.703 (0.038)	0.505 (0.053)	0.79	0.729 (0.131)	0.426 (0.100)	0.78
EE4	0.769 (0.032)	0.408 (0.049)		0.633 (0.082)	0.400 (0.057)	
**Social recovery**						
SR1	0.788 (0.024)	0.379 (0.038)		0.663 (0.316)	0.343 (0.130)	
SR2	0.880 (0.019)	0.225 (0.034)	0.79	0.653 (0.270)	0.265 (0.161)	0.72
SR3	0.556 (0.037)	0.691 (0.041)		0.482 (0.386)	0.670 (0.133)	
**General well-being**						
GWB1	0.846 (0.017)	0.284 (0.028)		0.397 (0.604)	0.284 (0.028)	
GWB2	0.909 (0.012)	0.174 (0.021)	0.90	0.431 (1.017)	0.199 (0.068)	0.71
GWB3	0.856 (0.030)	0.267 (0.051)		0.521 (1.340)	0.251 (0.180)	
**Sleep quality**						
SQ1	0.854 (0.023)	0.271 (0.039)		0.673 (0.090)	0.321 (0.039)	
SQ2	0.815 (0.026)	0.336 (0.042)	0.79	0.873 (0.070)	0.250 (0.058)	0.80
SQ3	−0.559 (0.042)	0.687 (0.046)		−0.545 (0.054)	0.526 (0.047)	
**Being in shape**						
BIS1	0.731 (0.030)	0.446 (0.044)		0.515 (0.855)	0.324 (0.221)	
BIS2	0.725 (0.030)	0.474 (0.044)	0.80	0.358 (0.849)	0.467 (0.194)	0.54
BIS4	0.805 (0.026)	0.352 (0.042)		0.287 (0.332)	0.357 (0.053)	
**Personal accomplishment**						
PA2	0.726 (0.041)	0.472 (0.059)		0.727 (0.160)	0.428 (0.116)	
PA3	0.602 (0.042)	0.637 (0.050)	0.67	0.613 (0.160)	0.567 (0.116)	0.67
PA4	0.572 (0.045)	0.672 (0.052)		0.497 (0.132)	0.661 (0.067)	
**Self-efficacy**						
SE2	0.693 (0.034)	0.520 (0.048)		0.199 (0.287)	0.469 (0.115)	
SE3	0.813 (0.022)	0.339 (0.035)	0.83	0.481 (0.380)	0.315 (0.224)	0.71
SE4	0.840 (0.025)	0.294 (0.043)		0.824 (0.461)	0.131 (0.267)	

**TABLE 4:**
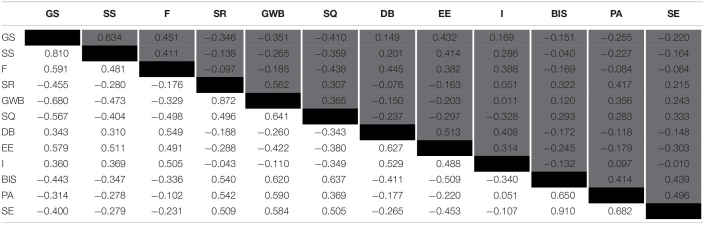
Standardized relationships between latent variables.

*White portion of the matrix reflects CFA standardized relationships. Gray portion of the matrix reflects ESEM standardized relationships.*

In terms of the relationships between RESTQ-Sport-36 subscales and mood states as measured by the Brief POMS, moderate to strong relationships exist between *General Stress, Social Stress, General Well-being, Sleep Quality, Emotional Exhaustion, Being in Shape*, and *Self-Efficacy* and all six mood states. Weak correlations were observed between *Fatigue, Disturbed Breaks*, and *Injury* and mood states like *Anger, Vigor, Depression*, and *Confusion.* Of the mood states, *Tension* and *Fatigue* shared moderate to strong correlations with most if not all of the RESTQ-Sport-36 subscales. Pearson correlation coefficients are reported in Table 5.

**TABLE 5:** Correlations between RESTQ−Sport−36 variables and Brief-POMS mood states.

	Tension	Anger	Fatigue	Vigor	Depression	Confusion
General stress	0.697	0.640	0.606	–0.435	0.777	0.616
Social stress	0.543	0.725	0.487	–0.304	0.539	0.479
Fatigue	0.469	0.297	0.624	–0.260	0.401	0.382
Social recovery	–0.361	–0.337	–0.339	0.464	–0.402	–0.284
General wellbeing	–0.490	–0.518	–0.420	0.561	–0.555	–0.456
Sleep quality	–0.542	–0.400	–0.560	0.388	–0.435	–0.447
Disturbed breaks	0.346	0.252	0.393	–0.210	0.274	0.343
Emotional exhaustion	0.444	0.482	0.503	–0.312	0.529	0.408
Injury	0.289	0.192	0.503	–0.068	0.187	0.194
Being in shape	–0.360	–0.326	–0.462	0.489	–0.329	–0.384
Personal accomplishment	–0.308	–0.379	–0.194	0.339	–0.293	–0.320
Self-efficacy	–0.362	–0.317	–0.339	0.432	–0.347	–0.421

*n = 304.*

## Discussion

Within a robust United States collegiate student-athlete sample, the purposes of this study were to: (a) examine the reliability and structural validity of the RESTQ-Sport-36 using SEM procedures and (b) examine construct validity of the RESTQ-Sport-36 via correlations between its subscales and Brief-POMS mood states. An additional, applied purpose of the study was to determine best scoring methods for the RESTQ-Sport-36. Results of the current study indicate that the 12-factor, first order model is the most parsimonious model, and thus interpretation of higher order factor scores may prove less meaningful than individual subscale scores in this population. Results also demonstrated initial support for the reliability of RESTQ-Sport-36 subscales. Additionally, each of the RESTQ-Sport-36 subscales were moderately or strongly correlated with mood states, thus providing evidence for the construct validity of the measure. Overall, the current findings provide convincing evidence in support of the RESTQ-Sport-36 use in the collegiate student-athlete population.

The findings generated from the current CFA and ESEM procedures are consistent with those reported previously (Kallus and Kellmann, 2016; Nicolas et al., 2019). The relationships between latent constructs were also consistent with those reported by Nicolas et al. (2019), whereby stress and recovery subscales are inversely related to one another. However, Nicolas et al. (2019) suggested that the hierarchical models showed a comparable fit to the first order model, thus supporting the adoption of hierarchical scoring methods in future research. By contrast, the current data suggest that the fit of both hierarchical models (Models 2 and 3) fell below standards of acceptability and were inferior to the Model 1 comparison in both CFA and ESEM scenarios. Taking in to account the entirety of the literature, using the 12 RESTQ-Sport subscales poses minimal risk for researchers and practitioners alike.

The current findings offer a notable contribution to the extant literature, in that CFA procedures may be too rigid to account for cross-loading items and/or the complexity of the model. Thus, an ESEM and/or Bayesian modeling procedure would be suitable for future psychometric evaluations of the RESTQ-Sport measures. As it relates to the ongoing development of the RESTQ-Sport-36, the current data prompt consideration regarding the unique contributions (e.g., cross-loadings, low factor loadings on scored factor) of each item and discriminant validity of the measurement model (e.g., high standardized relationships between select factors). Additionally, and in combination with the study by Nicolas et al. (2019), there is a need for additional research methods to ascertain the value of hierarchical factor scoring and interpretation.

Researchers have previously claimed that the POMS and the RESTQ-Sport are related measures (Kellmann and Kallus, 2001; Saw et al., 2016), a claim that was again supported by the current findings. Given the moderate to strong relationships observed between most of the RESTQ-Sport-36 subscales and mood states, it is worth considering how mood may play a role in the regulation of thoughts, information processing, and memory (Clore and Huntsinger, 2007; Storbeck and Clore, 2008). Within the occupational health literature, it has been suggested that mood repair is one of the primary functions of psychological recovery from work (Fuller et al., 2003; Sonnentag and Fritz, 2007). Further, mood dysregulation has been long associated with non-functional overreaching, overtraining, and burnout symptoms in athletes (Gould and Whitley, 2009; Meeusen et al., 2013). These data in the context of previous research collectively suggest that mood states and/or mood repair may be closely related to athlete levels of perceived stress and recovery. This phenomenon is important within applied contexts, as collegiate student-athlete mood states may have a significant impact on their psychological responses to training, competition, academic progress, and/or life events.

### Limitations and Directions for Future Research

The contributions of the current study to the extant literature notwithstanding, there are a number of limitations of the current methodology that prompt specific directions for future research. A considerable number of statistical analyses were performed using the same sample, and while the current findings expand on the psychometric properties of the RESTQ-Sport-36 in English-speaking populations, the generalizability of the findings is limited to primarily white/Caucasian collegiate student-athletes. Future research is warranted to explore the psychometric properties of the RESTQ-Sport-36, as well as the links between perceived stress, recovery, and mood states among samples of athletes varying by culture, ethnicities, nationalities, and competition levels. In addition, the current study did not draw direct connections between the athletes’ perceived stress and recovery experiences with behavioral antecedents or outcomes. Longitudinal research could involve RESTQ-Sport-36 monitoring alongside daily internal training load metrics such as training load volume and/or session rate of perceived exertion (sRPE) as an indicator of training load intensity. In turn, future research could be conducted to ascertain the recovery behaviors or activities completed by athletes, in order to optimize their stress-recovery balance. Specific to the dual-career demands experienced by collegiate student-athletes, future research should examine the RESTQ-Sport-36 in the context of student-athlete identity (Lupo et al., 2017a) and motivation (Lupo et al., 2017b). Future examinations of collegiate student-athlete stress and recovery experiences within broader theoretical frameworks (i.e., motivation, dual-career identity, burnout, etc.) would hold great importance in establishing the applied value of RESTQ-Sport-36 in this population.

### Conclusion

The purpose of the current study was to examine the reliability and validity of the RESTQ-Sport-36 for use in the collegiate student-athlete population. The current findings provide support for the measure’s use in this population, while also initiating pause as it relates to the scoring and interpretation of hierarchical factors such as *Total Stress* and *Total Recovery.* Overall, the RESTQ-Sport-36 may add value to existing collegiate student-athlete training load and competition monitoring protocols.

## Data Availability Statement

The raw data supporting the conclusions of this article will be made available by the authors, without undue reservation.

## Ethics Statement

The studies involving human participants were reviewed and approved by University of Wisconsin-Milwaukee Institutional Review Board. The patients/participants provided their written informed consent to participate in this study.

## Author Contributions

All authors listed have made a substantial, direct and intellectual contribution to the work, and approved it for publication.

## Conflict of Interest

The authors declare that the research was conducted in the absence of any commercial or financial relationships that could be construed as a potential conflict of interest.
